# Preconception Maternal Mentoring for Improved Fetal Growth among Indonesian Women: Results from a Cluster Randomized Controlled Trial

**DOI:** 10.3390/nu15214579

**Published:** 2023-10-28

**Authors:** Hamam Hadi, Siti Nurunniyah, Joel Gittelsohn, Ratih Devi Alfiana, Fatimatasari, Emma C. Lewis, Detty Nurdiati

**Affiliations:** 1 Alma Ata Graduate School of Public Health, The University of Alma Ata, Yogyakarta 55183, Indonesia; 2 Alma Ata Center for Healthy Life and Foods (ACHEAF), The University of Alma Ata, Yogyakarta 55183, Indonesia; 3 Department of Midwifery, Faculty of Health Sciences, The University of Alma Ata, Yogyakarta 55183, Indonesia; nurunniyah.siti@almaata.ac.id (S.N.); ratihdevi@almaata.ac.id (R.D.A.); fatimatasari@almaata.ac.id (F.); 4 Human Nutrition, Department of International Health, Johns Hopkins University Bloomberg School of Public Health, Baltimore, MD 21205, USA; jgittel1@jhu.edu (J.G.); elewis40@jhu.edu (E.C.L.); 5 Department of Obstetrics & Gynecology, Faculty of Medicine, Public Health, and Nursing, Universitas Gadjah Mada, Yogyakarta 55281, Indonesia; dnurdiati@yahoo.com

**Keywords:** maternal mentoring, preconception, fetal growth, maternal and child health, Indonesia

## Abstract

The prevalence of stunting in young children is associated with poor growth during the prenatal and early postnatal periods. A maternal mentoring program was developed for Indonesian women to improve birth outcomes. A cluster-randomized controlled trial (CRCT) was conducted in three sub-districts of the Special Region of Yogyakarta, Indonesia. A total of 384 eligible participants were randomly allocated to either an intervention (received the maternal mentoring program and standard care; *n* = 189) or control (received standard care only; *n* = 195) group. The maternal mentoring program provided preconception health education; health monitoring; and text message reminders for preconception women. Fetal growth was measured between gestational weeks 27 and 30 using the estimated fetal weight generated from ultrasonographic measurements. Birth weight was measured within 24 h of birth. A structured questionnaire captured women’s demographics, pregnancy readiness, and body mass indexes (BMIs). After adjustment, fetal weight was 14% (95% CI: 5.1–23.0) higher in the intervention group than in the control group, and the average weight-for-length Z-score at birth was 0.16 (95% CI: 0.04–0.30) higher in the intervention group than in the control group. The maternal mentoring program was associated with improved fetal growth and birth weight in this population and should be considered for scale-up to other settings, nationally and globally.

## 1. Introduction

Maternal and child health is a pressing health challenge worldwide. Restricted linear growth in particular presents major public health implications for under-resourced communities in low- and middle-income countries (LMICs) [1,2,3]. In Indonesia, the maternal mortality rate (MMR) and infant mortality rate (IMR) have been on the decline but remain relatively higher than the 2015 Millennium Development Goals target of only 102 deaths per 100,000 live births [4,5,6]. In addition, impaired growth and development of children is especially concerning in developing countries, where the prevalence of stunting and wasting in children under 2 years of age is 29.1% and 6.3%, respectively [7]. In Indonesia, 21.6% of children are stunted, and 7.7% are wasted, surpassing the average among all developing countries [8].

Stunting can result from inadequate fetal growth, which is typically characterized by having a birth weight that does not match the gestational age at birth (referred to as ‘Small for Gestational Age (SGA)’). SGA babies born at term have a 2.43-times greater risk of stunting and a 2.52-times greater risk of wasting compared with normal-weight babies, and not surprisingly, those born preterm are at even greater risk (a 4.51-times greater risk of stunting and a 4.19-times greater risk of wasting) [9]. There are no data regarding the current prevalence of SGA in Indonesia, but it is estimated that approximately 6% of babies are born with a low birth weight (LBW) among those whose weights are recorded. Across all developing countries, estimates are higher, reaching nearly 27% of all live births being considered SGA [10]. Importantly, exclusive breastfeeding has been found to play a significant role in protecting young children from stunting in under-resourced populations [11], but the prevalence of exclusive breastfeeding remains low in these settings [12,13].

Moreover, despite intervention approaches aimed at ensuring pregnant women receive adequate early prenatal care, many settings where birth outcomes are poor have not seen substantial improvements [14]. Thus, based on the literature and our understanding from previous formative research conducted in Indonesia, we believe that interventions to improve birth outcomes should begin at an earlier stage, prior to conception [14,15], during which healthcare providers may have greater opportunities to intervene and successfully improve outcomes for both the mother and baby.

Preconception health services, including maternal mentoring programs, typically involve a series of interventions aimed at identifying and modifying behaviors pertaining to biomedical and social risks associated with maternal and child health before, during, and after pregnancy [16,17]. Several studies conducted in other settings show that preconception health services can improve childbirth and pregnancy outcomes, thus reducing the likelihood of maternal and infant mortality [18,19]. Likewise, it is well documented that poor preconception health is associated with poor pregnancy outcomes [20,21].

Many women in under-resourced settings are not well equipped to adopt certain health-related behaviors prior to becoming pregnant. Relatedly, potential risks associated with poor pregnancy outcomes are more likely to arise in women who are unaware of these risks or their consequences and are often unprepared for pregnancy. Therefore, when made readily accessible, preconception health services can serve as a protective factor by providing pregnant women with adequate health services before their babies enter crucial stages of development [22].

To date, no preconception interventions have been developed and tested in Indonesia. Given the current state of maternal and child outcomes in this setting, there is a strong need for interventions that target preconception in Indonesian women. The present study sought to examine the impact of a maternal mentoring program provided to Indonesian women during preconception and pregnancy on fetal growth and birth weight [23].

## 2. Materials and Methods

The present study was conducted under the Community Alma Ata Partnership through the Updated Research and Education (CAPTURE) project. Detailed methods of this study have been reported elsewhere [24,25]. A cluster-randomized controlled trial (CRCT) design was used consisting of preconception women residing in either the Sedayu Subdistrict, Pajangan Subdistrict, or Pleret Subdistrict of the Special Region of Yogyakarta, Indonesia. Recruitment was conducted using national marriage registration data. Members of the research team met with preconception women and explained the research process, as well as the potential benefits and disadvantages of participating. If willing and able to participate, women were then asked to sign an informed consent form. The inclusion criteria included: (1) being a woman of childbearing age (but not currently pregnant) and currently married; (2) planning to remain in the research area for at least the next two years; and (3) giving informed consent. Women were excluded from this study if they (1) became pregnant at the beginning of this study; (2) anticipated moving in the next two years; or (3) planned to delay pregnancy. Of the total 1281 women recruited, 384 met the inclusion criteria and were willing to move forward with participation. The data were collected from this final sample via the CAPTURE data system from October 2018 to February 2021.

A total of 384 women were recruited within 122 identified clusters, and each was randomly allocated to either the intervention group (*n* = 189) or the control group (*n* = 195). Among them, 152 women in the intervention group and 158 in the control group became pregnant during the study period. We followed these pregnant women, and by February 2021, there were 113 newborns in the intervention group and 119 newborns in the control group who had their weights and lengths measured. Due to the COVID-19 pandemic, we did encounter some challenges in following the remaining women from our initial sample. Data were obtained using a questionnaire conducted at two time points: pre-test and post-test. Pre-tests occurred during the period before pregnancy, while post-tests occurred three weeks later. Clusters were randomly allocated to the treatment group using computer-generated random allocation [26]. Clusters 1–61 were assigned to the intervention group, and clusters 62–122 were assigned to the control group. Thus, the intervention and control groups each consisted of 61 clusters.

The mentors consisted of undergraduate students enrolled in the program areas of midwifery, nutrition, nursing, pharmacy, and hospital administration, and who had prior involvement in maternal and child health surveillance activities conducted under the CAPTURE project.

The maternal mentoring program delivered to the intervention group involved the following sequential components: (1) preconception health education delivered once during an initial home visit via face-to-face counseling in addition to providing a supplemental educational booklet; (2) monthly monitoring of pregnancy status via WhatsApp (WA) or basic Short Messaging Service (SMS), where women were asked to respond to the text prompt, “Have you experienced signs or symptoms of pregnancy such as late menstruation, nausea, vomiting, or others?”; and (3) once women reported experiencing signs of pregnancy, they were sent a text reminder to book their first antenatal care (ANC) visit immediately. Follow-up messages were sent every other day and included reminders to comply with routine provider visits and recommended iron supplementation. The control group received standard care based on current nationally recommended procedures for women of childbearing age.

The CAPTURE team trained mentors to ensure that they were competent in their role as preconception health counselors during the initial home visit phase of the program. In addition, a standardized preconception education booklet and worksheet were used to ensure intervention quality and validity [27].

Fetal weight between weeks 27 to 30 was estimated using ultrasonography (USG) examination conducted by trained obstetricians. Each pregnant woman in the study sample was scheduled to visit an approved obstetrician once she entered the 27th week of gestation. Birth weight and birth length were measured within 24 h of the newborn’s birth by a trained midwife or nurse in the health clinic or hospital in which the delivery took place. Weight was measured using a digital weighing scale (SECA 876, Hannover, Germany) to the nearest 100 g, while length was measured using SECA measuring tapes to the nearest millimeter. A weight-for-length Z-score, length-for-age Z-score, and weight-for-age Z-score were generated using Stata 15 [28] for each newborn.

All data were then analyzed using Stata 15, which involved constructing a frequency distribution to determine participant characteristics, as well as bivariate and multivariable analyses. The average difference test between treatment groups was performed using chi-square and independent t-tests. Multilevel mixed-effects linear regression was used to explore the effect of the intervention on fetal growth and infant birthweight, with the factors of cluster, age, parity, level of education, employment status, income, and maternal body mass index (BMI) controlled for.

## 3. Results

There were no significant differences detected in socioeconomic or demographic characteristics between the intervention and control groups (Table 1). The majority of participants were of a healthy reproductive age, were nulliparous, had less than 12 years of education, were employed, had an income equal to or below the regional minimum wage, and reported having spent less than six months preparing for pregnancy.

### 3.1. The Effect of Maternal Mentoring on Fetal Growth

The estimated fetal weight (EFW) at 27–30 weeks of gestational age was 245.5 g higher (*p* < 0.001) in the intervention group compared with the control group (Table 2). Likewise, the percentile of EFW was 17.7 points higher (*p* < 0.001) in the intervention group compared with the control group (Table 2). The birth weight of babies was found to be 78.8 g higher (*p* < 0.05) in the intervention group than in the control group (Table 2). Both WLZ and WAZ were significantly higher (*p* < 0.05) in the intervention group than in the control group (Table 2). No impact was seen in terms of birth length.

### 3.2. The Effect of Maternal Mentoring on Estimated Fetal Weight

Further analyses using cluster-adjusted multilevel mixed-effects linear regression were performed to examine the effect of maternal mentoring on the EFW percentile. In addition to adjusting for clusters, variables including maternal age, maternal education, maternal employment status, maternal monthly income, and maternal BMI were also adjusted for. Based on this, we determined that the EFW percentile was 14 points higher (95% CI: 5.1–23.0) in the intervention group than in the control group (Table 3).

### 3.3. The Effect of Maternal Mentoring on Newborn Birth Weight

After adjusting the multivariate models, we found a significant difference in birth weight between the intervention and control groups (Table 4). Accordingly, the mean newborn birth weight was 65.7 g higher (95% CI: 1.9–129.5) in the intervention group than in the control group adjusted for cluster, maternal age, maternal education, socioeconomic status, and maternal preconception BMI (Table 4).

### 3.4. The Effect of Maternal Mentoring on Newborn Weight-for-Length Z-Score

After adjusting the multivariate models, we found a significant difference in birth weight, but not in birth length, between the intervention and control groups (Table 4). Accordingly, the mean weight-for-length Z-score was 0.16 points higher (95% CI: 0.01–0.30) in the intervention group than in the control group adjusted for cluster, maternal age, maternal education, socioeconomic status, and maternal preconception BMI (Table 5).

### 3.5. The Effect of Maternal Mentoring on Newborn Birth Length

After adjusting the multivariate models, we could not find a significant difference in birth length between the intervention and control groups (Table 6). Using the same statistical modeling, we could not find a significant difference in newborn length-for-age Z-score and newborn weight-for-age Z-score (not shown).

## 4. Discussion

The preconception maternal mentoring program described herein is the only program of its kind to be delivered as an intervention in Indonesian women with an overall goal of improving maternal and child health [25]. In the present analysis, we found that receiving the maternal mentoring program during preconception and pregnancy significantly improved fetal growth and birth weight for newborns. We also found that the newborn weight-for-length Z-score (WLZ), but not the length-for-age Z-score (LAZ), was significantly higher for women who received the program compared with those who only received standard care.

A number of previous preconception interventions have been conducted to examine their impacts on maternal knowledge [18,29,30], maternal self-efficacy [31,32], maternal risk behaviors [29,30,32,33,34,35], and various birth outcomes [29,36]. Preconception nutritional supplementation in particular has been shown to improve newborn birth length [37] which is the strongest predictor of linear growth status and stunting in the first two years of life [38].

Although one study showed that preconception education and counseling did not significantly reduce low birth weight, the intervention materials used in the study were only delivered to women at one time point during the preconception stage [36]. Therefore, we posit that the effect of the materials was not strong enough in this instance to elicit a change in women’s risk behaviors. In addition, the study did not provide women with adequate information about complying with routine provider visits and recommended iron supplementation before and during pregnancy.

Herein, we reported that the maternal mentoring program in our study led to greater reported readiness for pregnancy (i.e., women felt more prepared for pregnancy, had more time for motherhood, had time to discuss their pregnancy with their partner, were more likely to consume recommended iron and folic acid supplementation, were more likely to maintain a healthy diet, were more likely to not smoke, were more likely to avoid over-the-counter and herbal drugs, were more likely to have health care insurance, were more able to manage stress, and were more likely to seek early detection of STDs) [24]. Preconception maternal mentoring improved the timing of first ANC visits, and women who received preconception maternal mentoring were three times more likely to have an earlier ANC visit than those who did not receive this mentoring [25]. This latter finding may help to explain, in part, the effect of the maternal mentoring program on improved fetal growth and birth weight in this study given that early ANC has been associated with improved birth outcomes.

We believe our intervention is unique from previous studies focused on providing maternal mentoring programs in other settings, as our intervention included a comprehensive package of educational materials and multiple time points for delivery from preconception to post-delivery. Meanwhile, many previous studies have only consisted of one single educational or counseling session [30,31,33,35,36], or have consisted of more than one session but without support for accessing routine provider visits or recommended iron supplementation before and during pregnancy [30,31,33].

In the present study, each woman in the intervention group received basic education during preconception, as well as monthly WhatsApp/SMS messages until pregnancy, and other message reminders every other day to comply with routine provider visits and recommended iron supplementation during pregnancy until delivery. This approach is comprehensive and could be considered a strength of our study. In addition, as many women have access to mobile devices, this approach is also likely to be feasible among the broader population of women in this setting and should be considered by healthcare providers and the national government given the ease and low cost of sending automated messages.

Importantly, a previous study found that monthly nutritional education delivered during the third trimester led to 60% higher weight gain, 20% higher birth weight, and 94% lower LBW for newborns [39]. Similarly, guided nutritional counseling delivered during pregnancy through home visits led to a 0.95kg higher average gestational weight gain and 0.26 kg higher average birth weight in one previous intervention [40]. Moreover, nutritional education for pregnant women has been shown to result in improved newborn birth weight, especially when spouses are involved in the education. In one particular study, newborn birth weight was 0.40 kg higher for the newborns of couples where both partners received nutritional education compared with only the woman [41]. Each of these nutritional education sessions was delivered at least three times during either a home visit or counseling. Therefore, it is possible that the intensity and frequency of nutritional education and counseling sessions play an important role in the success of behavioral change interventions aiming to improve birth outcomes.

Additionally, there is a lack of evidence to show that maternal mentoring significantly impacts newborn birth length. In our study, the newborn length-for-age Z-score was not found to be different between the intervention and control groups. However, one multi-country randomized controlled trial conducted in rural and semi-rural settings demonstrated that the newborn birth length-for-age Z-score was 0.19 points higher for women who received nutritional supplementation at least 3 months prior to conception compared with women who did not receive any nutritional intervention [37]. This higher LAZ, in turn, led to a significantly lower prevalence of stunting at six months and twenty-four months of age among the children whose mothers had received preconception nutritional intervention [38]. In the case of our study, it is possible that having greater health knowledge among those women who received maternal mentoring led to better eating practices prior to conception; however, we did not collect data on nutrient intake prior to conception. A takeaway from a multi-country study in relation to ours is that nutritional intervention approaches seemed to more strongly impact the newborn LAZ in settings with a higher prevalence of malnutrition [37].

Given that inadequate fetal growth is strongly associated with stunting in developing countries, including Indonesia, the present study provides strong support for implementing maternal mentoring in addition to standard care for preconception and pregnant women in this setting. It is important to note that this study sample was obtained from women in Yogyakarta, who mostly have mobile devices, and may not be representative of all women across rural and urban regions of Indonesia; therefore, future research should explore the impact of such programs in broader populations in order to successfully reach the national goals for reduced stunting and wasting among children.

## 5. Conclusions

To our knowledge, this is the first intervention of its kind to investigate the impact of preconception maternal mentoring on fetal growth and birth weight outcomes in Indonesia, where child stunting and wasting remain common. Our findings demonstrate that a comprehensive maternal mentoring program provided in addition to standard care procedures may be feasible and effective for improving fetal growth and increasing birth weight, especially among a particularly under-resourced population of women. Future efforts are needed on behalf of researchers, healthcare providers, and policymakers to determine how maternal mentoring programs such as the one described herein can be adapted and scaled to improve outcomes for both mothers and children across Indonesia and worldwide.

## Figures and Tables

**Table 1:** Participant characteristics at baseline.

	Intervention Group	Control Group	
Variable	Total (*n* = 189) (%)	Total (*n* = 195) (%)	* *p*-Value
Age			
<20	6 (3.2)	15 (7.7)	0.15
20–35	176 (93.1)	174 (89.2)	
>35	7 (3.7)	6 (3.1)	
Parity			
Nulliparous	182 (96.2)	190 (97.4)	0.52
Multiparous	7 (3.8)	5 (2.6)	
Education level			
≤12 years	153 (81)	166 (85.1)	0.28
>12 years	36 (19)	29 (14.9)	
Employment status			
Yes	140 (74.1)	139 (71.3)	0.54
No	49 (25.9)	56 (28.7)	
Income			
≤Regional income	143 (75.7)	142 (72.8)	0.53
>Regional income	46 (24.3)	53 (27.2)	
Time for pregnancy preparation			
≤6 months	153 (81)	167 (85.6)	0.22
>6 months	36 (19)	28 (14.4)	
Maternal body mass index			
<18.5	28 (14.8)	35 (17.9)	0.06
18.5–24.5	117 (61.9)	133 (68.2)	
>24.5	44 (23.3)	27 (13.8)	

* *p*-value from the chi-squared/Fisher’s exact test.

**Table 2:** Fetal and newborn anthropometric characteristics.

Study Groups			
Fetal Anthropometric Characteristics	Intervention, *n* = 113	Control, *n* = 119	* *p*-Value
Estimated fetal weight/EFW (g), mean (SD)	1415.8 (443.3)	1170.3 (226.8)	<0.001
Percentile of EFW, mean (SD)	57.7 (34.5)	40.0 (26.6)	<0.001
	Intervention, *n* = 129	Control, *n* = 123	
Newborn birth weight (g), mean (SD)	3117.8 (277.5)	3039.0 (264.7)	<0.05
WLZ score, mean (SD)	−0.3 (0.7)	−0.5 (0.7)	<0.05
WAZ score, mean (SD)	−0.4 (0.6)	−0.5 (0.6)	<0.05
Newborn length (cm), mean (SD)	49.2 (1.2)	49.1 (1.1)	>0.05
LAZ score, mean (SD)	−0.2 (0.7)	−0.2 (0.6)	>0.05

EBW was estimated using USG measurements. EBW percentile was calculated based on intergrowth. WLZ score was calculated based on intergrowth standard. WAZ score was calculated based on intergrowth standard. * *p*-value was obtained from independent *t*-test.

**Table 3:** Effect of maternal mentoring on percentile of estimated fetal weight.

EFW Percentile	Cluster-Adjusted Multilevel Mixed-Effects Linear Regression		
	Coefficient	SE	(95% CI)
Study group			
Intervention	14.0	4.6	5.1–23.0
Control	-	-	-
Age (years)			
<20	4.4	7.9	−11.2–20.0
20–35	-	-	-
>35	−6.2	12.9	−31.5–19.1
Maternal education			
≤12 years	−13.7	5.5	−24.5–(−3.0)
>12 years	-	-	-
Employment status			
Employed	-	-	-
Unemployed	−4.47	4.4	−13.2–4.2
Maternal monthly income			
<national wage	-	-	-
≥national wage	−5.74	4.8	−15.0–3.6
Maternal body mass index			
Underweight	−4.4	5.5	−15.3–6.4
Normal	-	-	-
Overweight	−10.5	5.4	−21.3–0.15

Regression coefficients, SE, and 95% CI were generated from multilevel mixed-effects linear regression adjusting for cluster, age, maternal education, employment status, maternal monthly income, and BMI (*n* cluster = 106).

**Table 4:** Effect of maternal mentoring on newborn birth weight.

Newborn Birth Weight	Cluster-Adjusted Multilevel Mixed-Effects Linear Regression		
	Coefficient	SE	(95% CI)
Study group			
Intervention	65.7	32.52	1.9–129.5
Control	-	-	-
Age (years)			
<20	−12.7	63.9	−137.8–112.5
20–35	−32.6	116.6	−261.1–195.9
>35			
Maternal education			
≤12 years	−52.3	42.0	−134.6–30.1
>12 years	-	-	-
Employment status			
Employed	39.2	35.6	−30.7–108.9
Unemployed	-	-	-
Maternal monthly income			
<national wage	-	-	-
≥national wage	−27.6	38.5	−103.1–47.8
Maternal body mass index			
Underweight	57.4	42.9	−26.8–141.6
Normal	-	-	-
Overweight	−8.8	44.7	−96.5–78.9

Regression coefficients, SE, and 95% CI were generated from multilevel mixed-effects linear regression adjusting for cluster, age, maternal education, employment status, maternal monthly income, and BMI (*n* cluster = 106).

**Table 5:** Effect of maternal mentoring on newborn weight-for-length Z-score.

Weight-for-Length Z-Score	Cluster-Adjusted Multilevel Mixed-Effects Linear Regression		
	Coefficient	SE	(95% CI)
Study group			
Intervention	0.162	0.08	0.04–0.3
Control	-	-	-
Maternal age (years)			
<20	4.4	7.9	−11.2–20.0
20–35	-	-	-
>35	−6.2	12.9	−31.6–19.1
Maternal education			
≤12 years	−13.7	5.45	−24.5–(−3.0)
>12 years	-	-	-
Maternal employment status			
Employed	−4.5	4.44	−13.2–4.2
Unemployed	-	-	-
Maternal monthly income			
<national minimum wage	-	-	-
≥national minimum wage	−5.7	4.76	−15.09–3.59
Maternal body mass index			
Underweight	0.11	5.53	−0.10–0.3
Normal	-	-	-
Overweight	−0.22	−5.46	0.08–0.4

Regression coefficients, SE, and 95% CI were generated from multilevel mixed-effects linear regression adjusting for cluster, age, maternal education, employment status, maternal monthly income, and BMI.

**Table 6:** Effect of maternal mentoring on newborn birth length.

Newborn Birth Length	Cluster-Adjusted Multilevel Mixed-Effects Linear Regression		
	Coefficient	SE	(95% CI)
Study group			
Intervention	0.08	0.14	−0.19–0.35
Control	-	-	-
Maternal age (years)			
<20	−0.26	0.27	−0.79–0.26
20–35	-	-	-
>35	−0.24	0.49	−1.2–0.72
Maternal education			
≤12 years	−0.15	0.18	−24.5–(−3.0)
>12 years	-	-	-
Maternal employment status			
Employed	−4.5	4.44	−0.50–0.20
Unemployed	-	-	-
Maternal monthly income			
<national minimum wage	-	-	-
≥national minimum wage	0.21	0.15	−0.09–0.50
Maternal body mass index			
Underweight	0.06	0.18	−0.29–0.41
Normal	-	-	-
Overweight	−0.15	0.19	−0.51–0.22

Regression coefficients, SE, 95% CI were generated from multilevel mixed-effects linear regression adjusting for cluster, age, maternal education, employment status, maternal monthly income, and BMI.

## Data Availability

The authors are very pleased to share the research data used in this manuscript.
